# Trapping Layers Prevent Dopant Segregation and Enable Remote Doping of Templated Self-Assembled InGaAs Nanowires

**DOI:** 10.1021/acs.nanolett.3c00281

**Published:** 2023-07-04

**Authors:** Chunyi Huang, Didem Dede, Nicholas Morgan, Valerio Piazza, Xiaobing Hu, Anna Fontcuberta i Morral, Lincoln J. Lauhon

**Affiliations:** † Department of Materials Science and Engineering, Northwestern University, Evanston, Illinois 60208, United States; ‡ Laboratory of Semiconductor Materials, Institute of Materials, EPFL, Route Cantonale, Lausanne, Vaud 1015, Switzerland; § The NUANCE Center, Northwestern University, Evanston, Illinois 60208, United States

**Keywords:** nanowire growth, remote doping, atom probe tomography, modeling

## Abstract

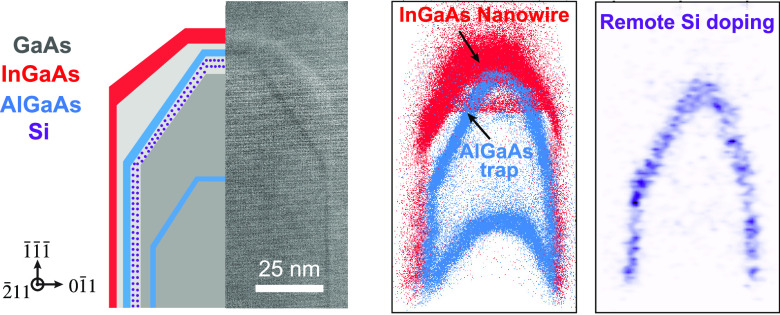

Selective area epitaxy is a promising approach to define nanowire networks for topological quantum computing. However, it is challenging to concurrently engineer nanowire morphology, for carrier confinement, and precision doping, to tune carrier density. We report a strategy to promote Si dopant incorporation and suppress dopant diffusion in remote doped InGaAs nanowires templated by GaAs nanomembrane networks. Growth of a dilute AlGaAs layer following doping of the GaAs nanomembrane induces incorporation of Si that otherwise segregates to the growth surface, enabling precise control of the spacing between the Si donors and the undoped InGaAs channel; a simple model captures the influence of Al on the Si incorporation rate. Finite element modeling confirms that a high electron density is produced in the channel.

The confinement of electrons in one-dimensional conduction channels enables future generations of nanoelectronic and optoelectronic devices including field effect transistors (FETs),^1−5^ thermoelectric devices,^6−9^ photo emitters and detectors,^10−16^ lasers,^17−19^ and devices for topological quantum computing.^20−25^ The performance of all such devices benefits from optimization of the charge carrier mobility, and applications that exploit quantum coherence in particular demand that scattering be minimized. In planar heterostructures, modulation doping is an effective strategy to achieve high free-carrier concentrations while limiting scattering by spatially separating ionized dopants from the channel.^26−29^ Modulation doping has been demonstrated previously in free-standing core–shell nanowires.^1,26,27,30−33^ However, it is challenging to integrate such free-standing nanowires into connected networks, particularly when it is necessary to preserve phase-coherence across junctions as for nanowire-based implementations of quantum computing.^20,34−36^

Template-assisted growth of nanowire networks by epitaxy methods^25,37−42^ is a promising approach to define pristine junctions between quantum wires. By patterning openings in an oxide mask using lithography, networks of GaAs nanomembranes with three-way junctions are selectively grown along defined orientations (Figure 1(a,b)). The GaAs nanomembranes support the growth of InGaAs nanowires at the apex of the nanomembrane under growth conditions that enable In and Ga to diffuse on the nanomembrane surface to the apex. It is challenging to simultaneously control the distribution of dopants in membrane-supported nanowire networks to realize modulation doping, which is necessary to achieve both high carrier concentration and high mobility. While modulation doping processes for planar heterostructures exist,^43−45^ they must be adapted in a way that preserves the surface mobility of group-III species while preventing dopant segregation or diffusion.^46^ In our earlier work,^24^ atom probe tomography (APT) analysis showed that Si doping concurrent with InGaAs nanowire growth leads to Si segregation on the (111) growth surface and selective doping of the upper nanowire surface. More recently, Si was incorporated in the GaAs nanomembrane to achieve quasi-remote doping of the InGaSAs nanowire region,^25^ but significant dopant concentrations remained at the interface of the GaAs membrane and InGaAs nanowire. Independent control of remote dopant distribution and nanowire morphology in supported networks has not previously been achieved.

**Figure 1:**
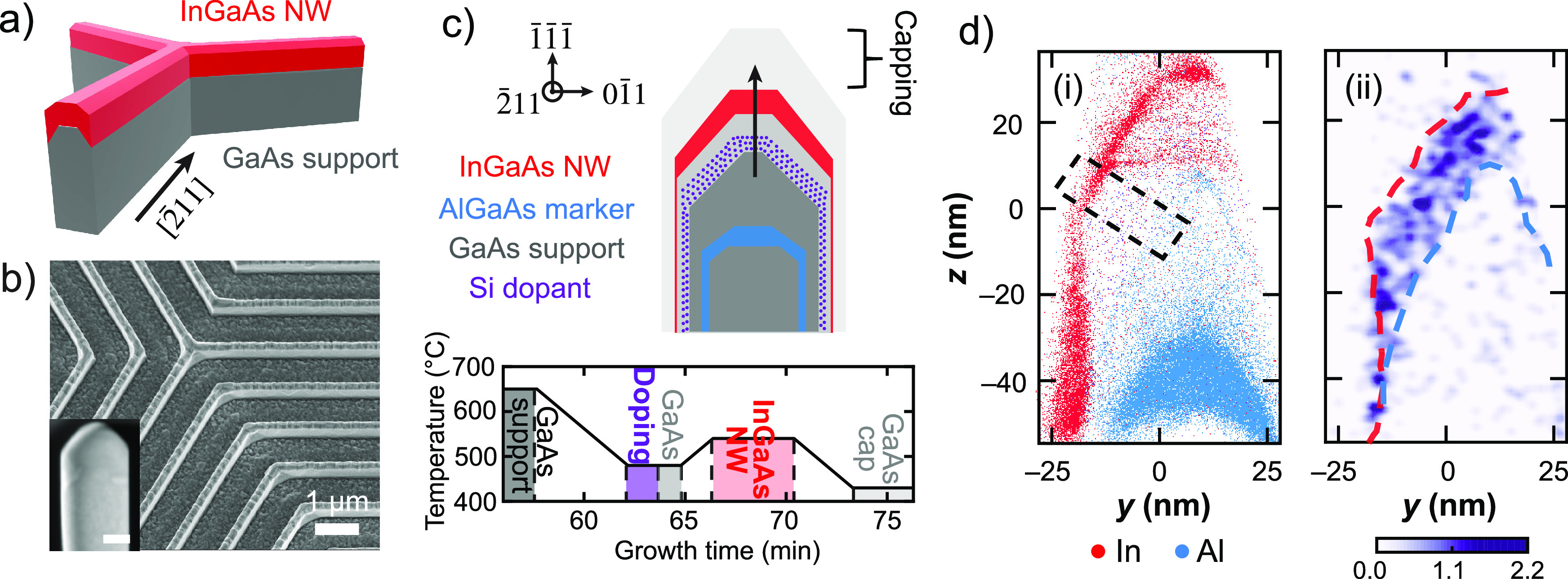
Growth of supported nanowire network and structure of Sample-1. a) Schematic of branched InGaAs nanowire atop a GaAs nanomembrane support. b) 20° tilted-view scanning electron microscopy (SEM) image of the branched nanowire/nanomembrane. Inset: Cross-section HAADF image of a single branch. Scale bar is 70 nm. c) Cross-sectional schematic of Sample-1. Bottom: temperature during the growth process for the region indicated by the black arrow in the schematic. d) (i) APT reconstruction of Sample-1 with In and Al rendered as red and blue dots, respectively. (ii) Si concentration (in units of 10^19^ cm^–3^) in the same region as (i). The blue and red dashed lines show the position of the dilute Al band and the outline of InGaAs nanowire (0.5% In), respectively.

Here we report a strategy for modulation doping of supported InGaAs nanowires templated by GaAs nanomembrane networks on GaAs(111)B substrates. Employing APT, we find that the growth of a thin AlGaAs layer following low-temperature Si doping prevents Si segregation at the growth interface and enables the subsequent higher temperature self-assembled growth of InGaAs nanowires. An empirical model of the Si partition coefficient relates the Si incorporation rate to the Al concentration. Finite element simulations of the electron distribution show that electrons donated by Si dopants are transferred to the InGaAs quantum wire and are localized away from the nanomembrane/nanowire interface. The demonstration of an approach to selectively localize dopants while maintaining an optimized nanowire morphology represents an important step toward quantum wires with mobilities and scattering rates that support new quantum applications.

Our approach to concurrent control of nanowire morphology and remote doping is illustrated in Figure 1, in which networks of epitaxial GaAs nanomembrane supports with controlled faceting direct the self-assembly of InGaAs nanowires (Figure 1(a,b)). Detailed growth conditions are described in Supporting Information Section S1. The supports are initially defined by selective area growth (SAG) in the openings of SiO_2_ masks at 630 °C, which provides good selectivity and enables control of the nanomembrane faceting with the Ga flux.^47^ A three-way nanowire junction is thereby defined, as shown at the center of Figure 1(b). A schematic cross-section of the nanowire and the nanowire growth sequence for Sample-1 are shown in Figure 1(c). We note that the roughness observed in Figure 1(b) is a result of the low-temperature protective capping layer and does not reflect the InGaAs nanowire morphology. The Ga shutter is closed, and the As shutter is open, while changing temperature to limit growth and prevent desorption of Ga. Thin Al_0.05_Ga_0.95_As marker layers are introduced at selected times to enable the growth interface shape to be reconstructed by STEM imaging (Figure S1) and APT (Figure 1(d)) after growth. Mapping of the location and shape of the growth interface at different times and under different conditions reveals how the facet growth rates and sizes change with growth conditions.

At the optimized temperature for growth of the GaAs support (630 °C), Si doping profiles in GaAs are substantially broadened by diffusion and/or surface segregation as observed in planar MBE growth studies^48,49^ and our prior work on similar nanowires.^24^ Because Si diffusion has been shown to be negligible in GaAs films grown by MBE below 500 °C,^50−52^ our approach to remote doping in Sample-1 was to lower the temperature to 480 °C prior to introducing a Si dopant layer of ∼5 nm in the GaAs support (Figure 1(c)). Subsequently, a spacer layer of nominally undoped GaAs was grown to separate the doped region from the InGaAs nanowire. The temperature was then increased prior to the selective growth of the InGaAs nanowire based on conditions optimized in prior work.^25^ A low-temperature GaAs capping layer with an additional AlGaAs marker layer was grown to protect the nanowire region during subsequent analysis.

Figure 1(d) presents a subset of the APT reconstruction of Sample-1 corresponding to a portion of the schematic in Figure 1(c) representing the nominal nanowire cross-section. The full reconstruction can be found in Supporting Information Section 2 along with the sample preparation and analysis conditions. Comparison of the schematic with the actual structure (Figure 1(d-i)) reveals several interesting deviations. First, Al is present beyond the AlGaAs marker layer region at the bottom of the reconstruction. We conclude that trace amounts of Al remain on the growth surface until the beginning of the growth of the Si-doped region, at which point the remaining Al is incorporated in a faint band visible in Figure 1(d-i). Second, the nominally undoped GaAs spacer is heavily doped with Si (Figure 1(d-ii)), despite being grown at a temperature at which Si is not expected to diffuse.

As noted above, (bulk) Si diffusion is reported to be negligible in GaAs films grown by MBE below 500 °C.^52^ However, we note that the As-rich growth environment that was used to achieve the desired nanowire faceting is known to promote Si diffusion.^53^ In addition, we observe that diffuse Si doping could result from Si segregation at the growth interface, leading to a surface excess that is gradually depleted after the Si flux is terminated. In such a case, differences in the dopant incorporation rates between distinct facets would drive surface diffusion.

To promote the rapid incorporation of Si at the intended position, we modified the growth process to include an additional AlGaAs layer immediately following the Si doping step (Sample-2, Figures 2, S3, and S4). The AlGaAs layer was followed by the growth of a GaAs spacer layer at the same temperature, prior to increasing the growth temperature for InGaAs nanowire growth as in the previous sample. As shown in Figure 2(b-ii), the Si is now distributed in a narrow band beneath the AlGaAs layer, indicating that Al promotes Si incorporation as intended. This approach was empirically motivated by the fact that Al modifies the surface energy of GaAs support facets and may therefore influence the diffusivity of Si and the relative preference for surface and bulk sites. Furthermore, Umansky et al. reported that a thin layer of GaAs could be successfully delta-doped by subsequently growing a GaAs-AlAs superlattice, which prevented Si segregation to the quantum well.^54^ Finally, we note that the dilute indium bands bisecting the supporting GaAs nanomembrane (Figure 2(b-i)) arise from stacking defects that are discussed in a later section.

**Figure 2:**
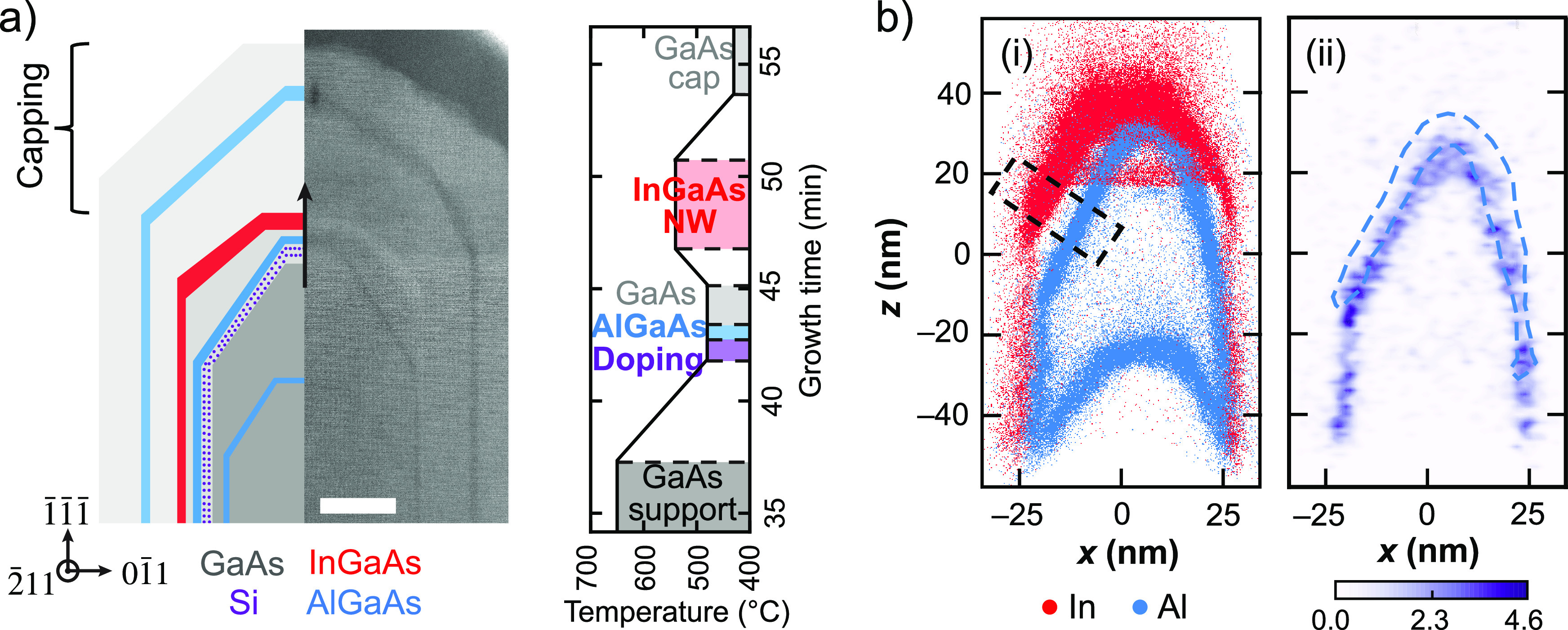
Dopant trapping scheme. a) Sample-2 schematic and growth temperature profile. The schematic is superposed with HAADF-STEM image showing InGaAs and AlGaAs layers as bright and dark bands, respectively. Scale bar is 25 nm. The first and third AlGaAs layers serve as fiducial markers, whereas the second AlGaAs layer is the dopant trap. The full HAADF-STEM image of the cross section, and the STEM-EDS image are shown in Figures S3 and S4. b) Reconstruction of nanomembrane supported nanowire showing (i) In and Al rendered as red and blue dots, respectively and (ii) Si concentration (in units of 10^19^ cm^–3^). The blue dashed line outlines the upper portion of the Al marker layer (2% Al). The Si concentration peaks beneath the Al marker layer.

To quantitatively analyze the Si concentration normal to the growth interface, Figure 3 plots the composition versus distance from the left-hand oblique facets of Sample-1 and Sample-2 as indicated by the dashed black boxes in Figures 1(d-i) and 2(b-i), respectively. In Sample-1 (Figure 3(a)), a peak in Al is observed following the onset of the low-temperature Si doping around −20 nm, indicating that excess Al becomes incorporated either due to the lowered growth temperature (480 °C) or the presence of Si, as discussed further below. Interestingly, the Si concentration builds very gradually after the Si shutter is opened, and then slowly begins to decrease when the shutter is closed. Clearly, the Si incorporation rate is not instantaneously governed by the direct vapor phase flux; additional mass transport pathways and/or surface segregation introduce a history dependence to the Si concentration. While the InGaAs nanowire is nominally undoped as intended, there is substantial Si in Sample-1 adjacent to the nanowire, which is not ideal as it would act as a source of impurity scattering. In Sample-2, the AlGaAs trapping layer results in a much narrower Si distibution (Figure 3(b)). The sequential appearance of the Si, Al, and In peaks with increasing distance (growth time) corresponds to the growth sequence. The onset of Si doping is indicated by the vertical purple line, indicating when the Si shutter was opened.

**Figure 3:**
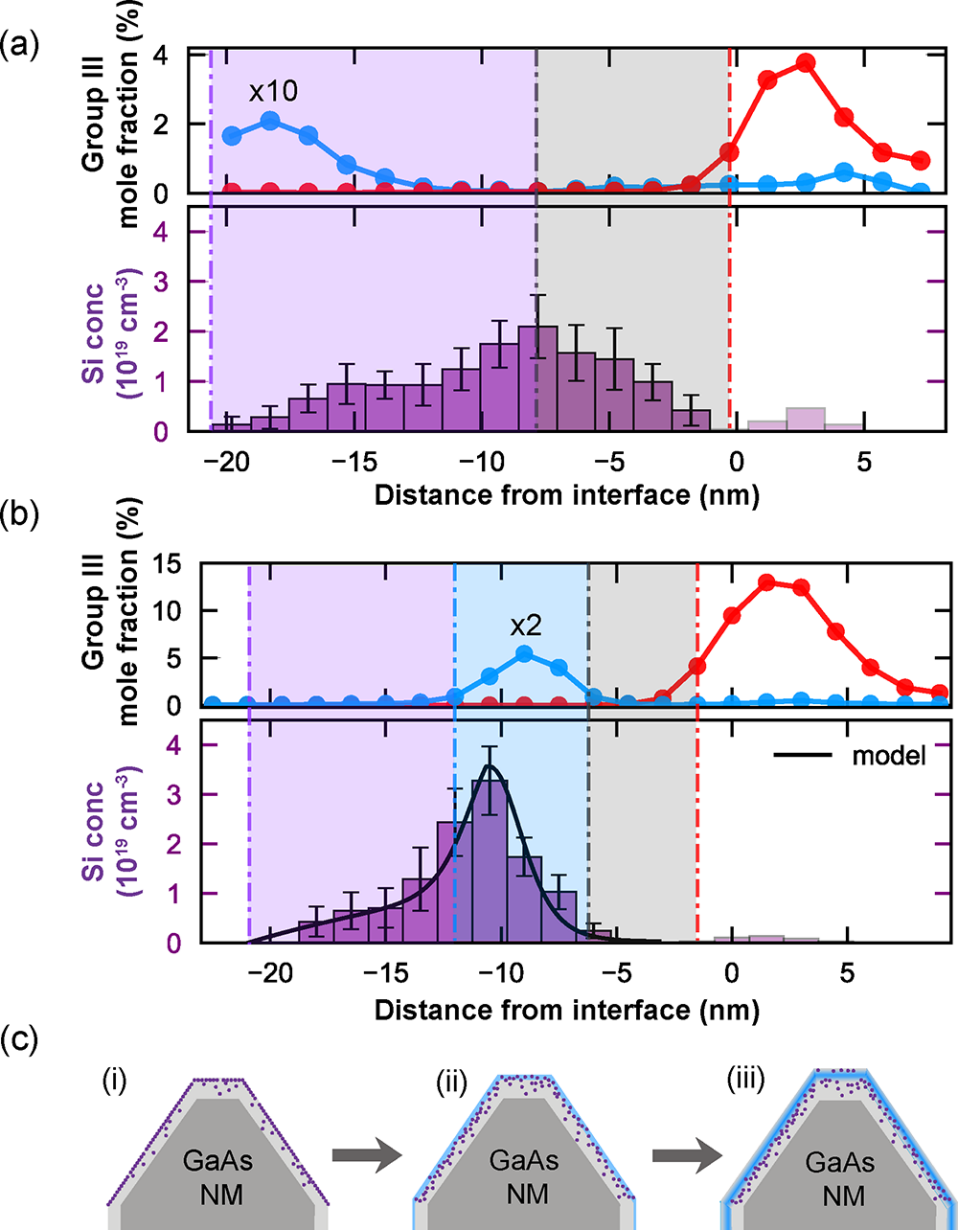
Si doping profiles without and with AlGaAs trapping layer. a) Si concentration and group III mole fraction (blue-Al; red-In) versus distance from the InGaAs nanowire interface on the left oblique facet of Sample-1 (Figure 1d). b) Composition profiles across the left oblique facet of Sample-2 along the growth direction (Figure 2b). The vertical red line indicates 6.5% In used to define the InGaAs nanowire surface. This surface was then evolved along the path of the steepest descent/ascent to generate composition profiles normal to the facet, and hence parallel to the growth direction. The purple, blue, and red vertical lines indicate where the Si, Al, and In shutters were opened, respectively. The vertical black lines indicate where the Si shutter was closed, and where Al was closed for Sample-2, defining the lower interface of the GaAs spacer. The position of the simultaneous Si shutter closing and Al shutter opening (blue line) is a parameter in the model used to generate the fit to the Si profile, as discussed below and in Supporting Information Section 3. The position of Al shutter closing (black line) was inferred assuming a constant growth rate at 480 °C for the doped region, trapping layer, and spacer. c) Schematic depiction of Si (purple dots) surface segregation followed by rapid incorporation with the introduction of Al (blue-shaded region).

The salient feature of Figure 3(b) is that the peak Si concentration is achieved after the Si flux is terminated, indicating the presence of excess Si at the growth interface that is incorporated after the Al flux is initiated. Under the growth conditions used here, bulk diffusion of In and Al is expected to be negligible, whereas surface diffusion of In along the nanomembrane sidewalls is necessary for nanowire formation on the upper facets.^55,56^ The fact that the lower interface of the InGaAs layer is more abrupt than the upper interface is consistent with this expectation; In on the sides of the nanomembrane continues to diffuse to the nanowire region after the In flux has been terminated. In contrast, the increase of the Si-doped layer is more gradual prior to the introduction of Al. We hypothesize that the onset is gradual because a substantial fraction of the Si remains at the growth interface, causing the surface concentration to increase with time, whereas the abrupt increase immediately followed by an abrupt decrease in Si concentration results from trapping by Al. Figure 3(c) presents a physical depiction of this hypothesis. When the Si flux is initiated by opening the Si shutter, excess Si begins to accumulate on the surface of the growing nanowire. As the surface Si concentration increases, there is a corresponding gradual increase in bulk incorporation (see the purple-shaded region in Figure 3(b)). When the Al shutter is opened (the blue-shaded region in Figure 3(b)), the Si begins to incorporate more rapidly, creating a spike in the Si concentration. As the excess surface Si becomes depleted, the incorporated Si concentration decreases rapidly, as observed in Figure 3(b) (purple to blue region) and depicted in Figure 3(c-ii) to (c-iii).

The incorporation of dopant atoms at the growth interface is driven by the difference in chemical potential between surface and bulk sites,^57−59^ influencing how effectively Si competes with Ga for incorporation on group III (Ga) sites. In a binary alloy such as GaAs, the addition of a third element to form a ternary compound may modify the incorporation of dopant species via one or more mechanisms. First, the third element (Al in this case) may change the bulk defect formation energy associated with dopant substitution^60−62^ by virtue of differences in size or bonding, which could increase or decrease the dopant incorporation rate. Second, an additional group III element such as In or Al will compete for binding sites, which could decrease the dopant incorporation rate. Third, Al may promote a change in the surface reconstruction, which could promote or inhibit dopant incorporation.^63,64^

Given that Al will modify the degree to which Si adatoms prefer surface versus bulk sites, and the fact that changes in the relevant chemical potentials can be linearized for sufficiently small perturbations in composition, we developed a simple empirical model that assumes the Si incorporation rate is proportional to the Si surface concentration, and that the incorporation rate increases linearly with Al surface concentration (see Supporting Information Section S3 for details). We represent these dependencies in an effective partition coefficient *k*, defined as the ratio between the bulk and surface solubility , which takes the form *k*(θ_Al_) = *c*_0_ + *c*_1_θ_Al_, where , and *c*_1_ captures the change in the partition coefficient due to the presence of Al. We then solve for the partition coefficient for GaAs and the Al-induced relative enhancement *k*/*c*_0_ that best describes the observed Si distribution (black line in Figure 3(b)). The fit in Figure 3(b) shows that the simple model captures the salient features of the observed Si distribution. The fit parameters also describe well the Si profile on the right-hand facet as shown in Figure S7.

The Al-induced enhancement of Si incorporation may arise from more than one mechanism, as noted above. One additional hypothesis to consider is that the initial Si increase results entirely from surface diffusion of Si from the nanomembrane sidewalls, rather than segregation of Si at the growth interface and that the decrease results not from the introduction of Al but from a depletion of the surface Si. As noted above, the In profile is definitely influenced by surface diffusion. However, it does not exhibit an abrupt jump, and the decay is more gradual. Hence, we conclude that surface segregation plays an important role in the Si incorporation kinetics and the observed dopant distribution.

Having demonstrated an effective approach to dopant localization, we now investigate the expected distribution of electrons with respect to the nanowire channel, the dopant atoms, and structural defects. We performed additional structural and chemical analyses using TEM and STEM to inform finite element simulations of the electron distribution based on the acquired structural information (Figure 4). The schematic in Figure 4(b) illustrates the formation of stacking faults just below the InGaAs nanowire. These defects appear as dark bands in the TEM image in Figure 4(a) and lead to streaking along [111] in the FFT of a HAADF-STEM image taken in the same region (Figure S5 shows FFT of the inset in Figure 4(c)). Because the stacking faults do not extend uniformly along the entire nanowire, the nanowire and capping region (region A, A′ in Figure 4(a,b)) are bicrystalline, with the two possible orientations indexed in the Figure S5. The GaAs support is single crystalline (region A in Figure 4(a)). A higher-resolution image showing the three distinct regions with corresponding diffraction patterns can be found in Figure S6. Note that in Figure 2(b-i) there are In lines beneath the AlGaAs trapping layer, even though In is introduced after that region was formed. This feature cannot result from simple bulk diffusion, which would produce a broad concentration profile, nor do we expect In to be incorporated preferentially at twin planes. Rather, we attribute the formation of sharp In lines to the boundaries between regions of different stacking and the associated dislocations that serve as rapid diffusion pathways.^65−67^ Atomistic simulations have shown that, in the vicinity of nanoscale twins, several types of dislocations may form, including classical Hall-Petch dislocations pinned by twin boundaries, partial dislocations traveling along twin planes, threading dislocations confined between neighboring twin boundaries, and jogged dislocations spanning multiple twins.^68,69^ The detailed analysis of the microstructure is beyond the scope of this work, which is instead focused on realizing modulation doping. Regardless of the detailed microstructure in the GaAs support, high mobilities can in principle be realized if the electrons donated by the Si dopants are localized well away from these defects, as we show below.

**Figure 4:**
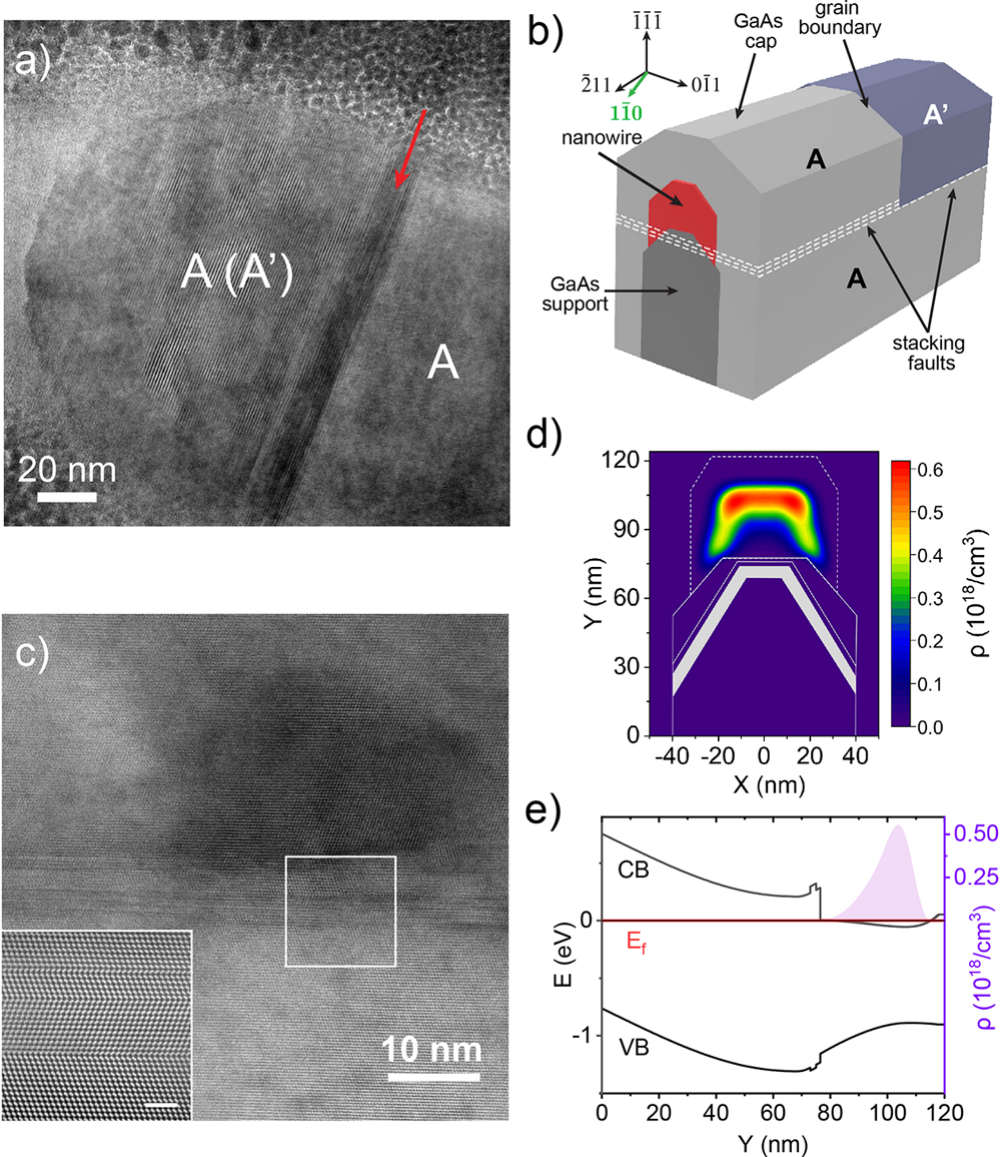
Analysis of electron distribution relative to structural features in Sample-2. a) Bright-field TEM of nanowire and capping region taken on [11̅0] zone axis. b) Perspective schematic of the cross-section showing grain boundary at termination of discontinuous stacking faults. The green arrow indicates the [11̅0] imaging direction. c) Annular bright-field (ABF)-STEM image (convergence and collection angles of 22 mrad and 11–22 mrad, respectively) showing defect band below InGaAs nanowire. Slight variations in thickness and/or tilt produce some of the large scale variations in contrast. Inset: High-resolution HAADF-STEM shows the presence of mirror twins. Scale bar is 2 nm. d) Electron density from finite element simulation using nextnano. e) Simulated conduction and valence band profiles.

Figure 4(d,e) shows the finite element simulations of the electron density and band structure for a GaAs-supported InGaAs nanowire in the optimal geometry for this approach, based on an earlier study,^25^ but with the optimized dopant placement achieved in the current study. Details of the simulations are described in Supporting Information Section 4 as well as carrier accumulation near the nanowire–nanomembrane interface (Figure S8). The key experimental advance reported here is the localization of the Si dopants with an AlGaAs layer to create a dopant-free spacer layer, which remains feasible at lower doping levels. Finally, the 2 nm AlGaAs layer and the thicker GaAs layer in the simulation are representative of the experimentally measured structure. The undoped band structure is given in Figure S9. The band-bending in the GaAs nanomembrane is created by the dopant layer. Although the AlGaAs layer creates a small barrier for electrons, robust electron transfer to the InGaAs nanowire is observed. The GaAs barrier layer is chosen to be thick enough to reduce impurity scattering while thin enough to enable electron transfer (see Figure S10 for further discussion). The band-bending in InGaAs results from inclusion of a realistic (i.e., previously measured) concentration gradient that creates a potential minimum well away from the nanowire–nanomembrane interface, as shown in Figure 4(e), leading to electron localization (Figure 4(d)) well away from the observed planar defects. Furthermore, the introduction of a surfactant such as Sb during growth could prevent the generation of these defects.

In summary, we have developed an integrated strategy to independently control the morphology and doping of templated InGaAs nanowire networks grown by MBE. Under low-temperature growth conditions that are effective for modulation doping in planar heterostructures, Si dopants segregate to the nonplanar nanowire growth interface, resulting in broadened doping profiles and the potential for impurity scattering. Insertion of a thin and dilute AlGaAs trapping layer between the doping region and GaAs spacer layer enables the complete incorporation of Si prior to nanowire growth. A simple empirical model establishes a quantitative correlation between the Al concentration and Si incorporation rate, motivating further experimental investigation and computational design of trapping layers.

## Supporting Information Available

Details of the sample growth, preparation, and analysis conditions; additional electron microscopy and atom probe tomography analysis; details of the dopant incorporation modeling and finite element modeling (PDF)
