# Supplementary material for: Trapping Layers Prevent Dopant Segregation and Enable Remote Doping of Templated Self-Assembled InGaAs Nanowires
Source: Nano Lett. 2023 Jul 4;23(14):6284–91. doi: 10.1021/acs.nanolett.3c00281 (PMC10375592; doi:10.1021/acs.nanolett.3c00281)
Supplement: Supplementary file 1 — nl3c00281_si_001.pdf [file nl3c00281_si_001.pdf]

Supporting Information for Trapping Layers Prevent Dopant Segregation and Enable Remote Doping of Templated Self-Assembled InGaAs Nanowires

Chunyi Huang,[†,§] Didem Dede,[‡,§] Nicholas Morgan,[‡] Valerio Piazza,[‡] Xiaobing Hu,[†,¶] Anna Fontcuberta i Morral,[*,‡] and Lincoln J. Lauhon[*,†]

†*Department of Materials Science and Engineering, Northwestern University, Evanston, Illinois 60208, United States*

‡*Laboratory of Semiconductor Materials, Institute of Materials, EPFL, Route Cantonale, Lausanne, VD, 1015, Switzerland*

¶*The NUANCE Center, Northwestern University, Evanston, Illinois60208, United States*

§*Contributed equally to this work*

E-mail: anna.fontcuberta-morral@epfl.ch; lauhon@northwestern.edu

S1 Sample growth

MBE growth was performed in a DCA P600 MBE chamber. For the selective area epitaxy, the fabrication steps are similar to those in our previous work.[1] 25 nm of SiO_2 was deposited on GaAs (111)B substrates using plasma-enhanced chemical vapor deposition (PECVD). Then the substrate was coated by 35 nm ZEP resist, patterned by electron beam lithography, and developed in cold n-amyl acetate. The pattern was transferred to the oxide layer by

dry etching employing CHF_3/SF_6 chemistry. Finally, the samples were etched for 10 s in a highly dilute buffered HF solution to remove oxide residues inside the patterned features and smoothen the mask surface. Prior to the growth, samples were degassed at a manipulator set temperature of 400 °C for 2 h. They were then transferred to the growth chamber and annealed further at 630 °C for 10 min. Following the annealing, GaAs supports were grown at the same temperature at an equivalent 2D GaAs growth rate of 1 Å/s with an arsenic BEP of 4×10^{-6} Torr. The Al flux used for the marker layer growth is much smaller than the Ga flux such that no perturbation of the growth rate is observed.

In both samples studied, the manipulator temperature was lowered to 480 °C for Si doping. Nominally undoped spacer layers of GaAs or AlGaAs/GaAs were grown at the same temperature. Samples were then heated to 540 °C for InAs nanowire growth, The In flux corresponded to an InAs equivalent growth rate of 0.3 Å/s, and the As BEP was increased to 7×10^{-6} Torr. The nanowire growth time was 4 min, and then the sample was then cooled for GaAs shell capping, which was performed at 430 °C at a Ga growth rate of 1 Å/s and As pressure of $\leq 1 \times 10^{-5}$ Torr. A thin marker AlGaAs marker layer was introduced during the shell growth, and then the sample was cooled down under an As flux.

The AlGaAs markers and the InGaAs nanowire are shown by HAADF-STEM in Figure S1.

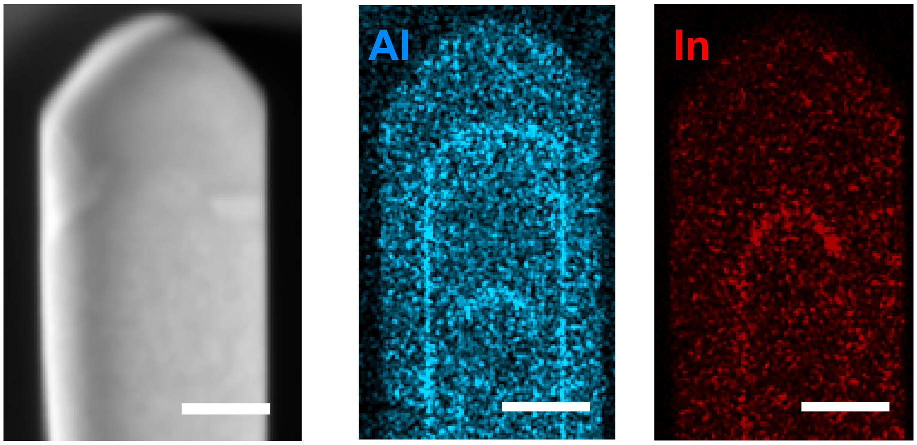

Fig. S1: HAADF-STEM (left) and EDS maps (mid, right) of a representative branch of Sample-1. Scale bars are 70 nm.

S2 Sample preparation and analysis conditions

Specimens for APT analysis were prepared using standard FIB lift-out and sharpening procedures[2,3] with the analysis direction perpendicular to (111) planes. The final specimens were sharp needle-shaped tips with diameters less than 100 nm. APT was performed using LEAP 5000XS (CAMECA, Madison, WI) with a 355 nm laser at 250 kHz pulse frequency, a 0.004-0.006 ions/pulse detection rate, a background temperature of 30 K, and laser pulse energies between 0.5 and 0.8 pJ. Reconstructions and further analysis were conducted using the commercial software package IVAS. The tip profile method was applied, in which the SEM images of the tips before analysis were imported to determine the reconstructed radius as a function of analyzed depth.

Sample-1 was analyzed by STEM using FEI Tecnai Osiris at 200kV. Sample-2 was also analyzed by aberration-corrected TEM and STEM using JEOL ARM200CF at 200 kV. Specimens for (S)TEM analysis were prepared using standard FIB lift-out and thinning/cleaning.[4,5]

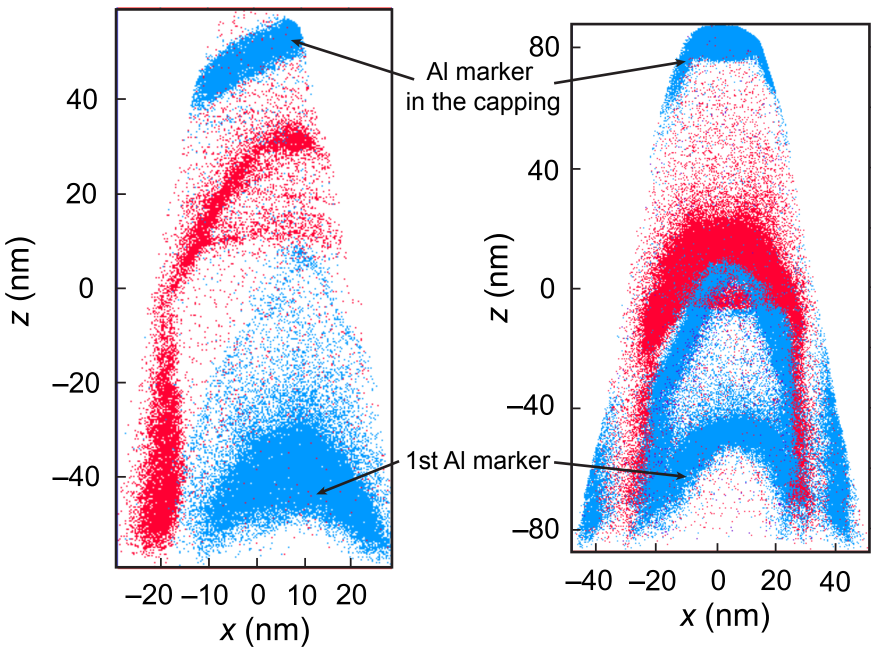

Fig. S2: Full APT reconstruction of (a) Sample-1 and (b) Sample-2, with the top Al marker and capping layer captured. In, Al and Si atoms are mapped in red, blue, purple dots respectively. Ga and As are not mapped here so that the dilute species are visible.

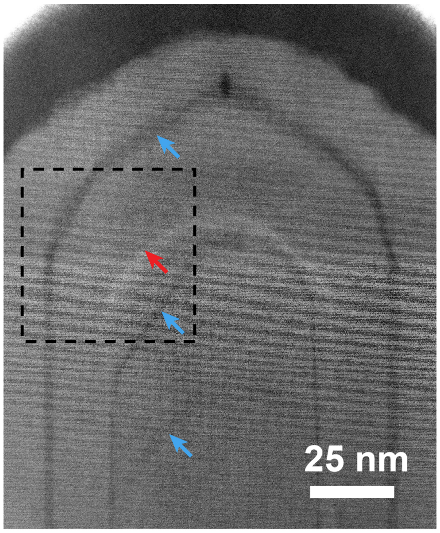

Fig. S3: HAADF-STEM image of the cross-section of Sample-2, showing the InGaAs nanowire (red arrow) and three AlGaAs layers (blue arrows).

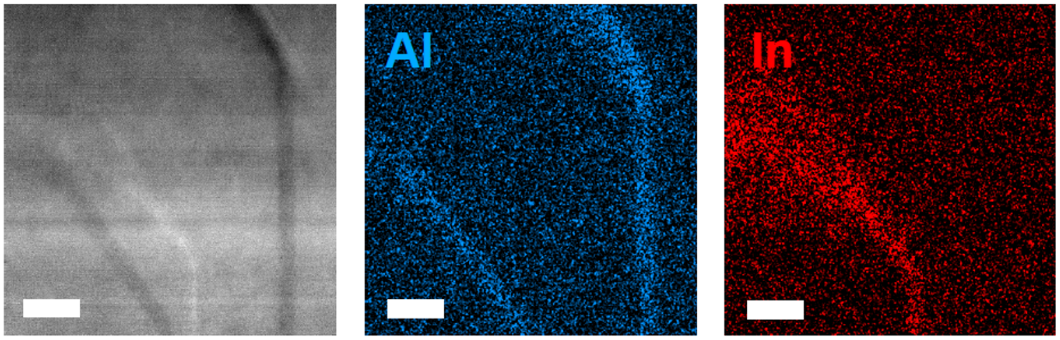

Fig. S4: HAADF-STEM (left) and EDS maps (middle, right) of another region of Sample-2. Scale bars are 10 nm.

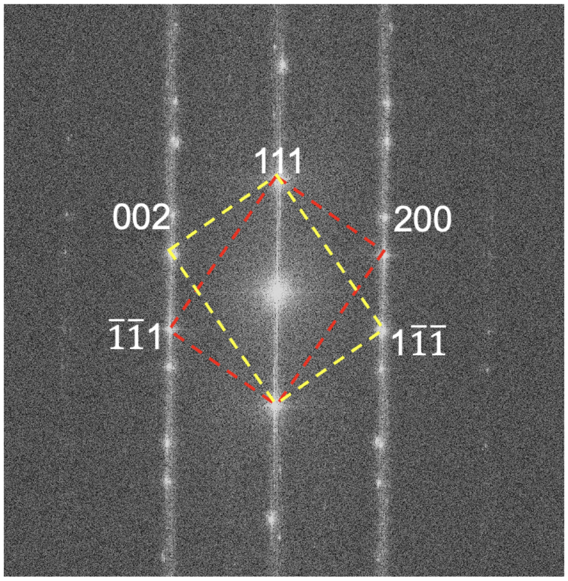

Fig. S5: FFT of the region near the bottom of the Sample-2 nanowire, indicating the presence of twinning and stacking faults.

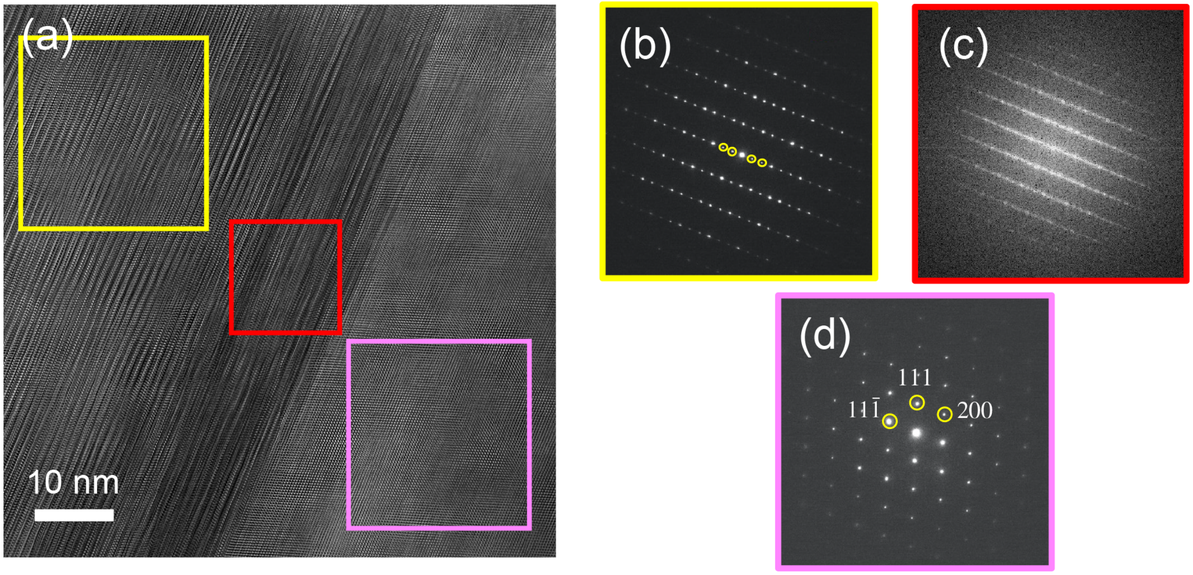

Fig. S6: (a) Bright-field TEM image of the top of Sample-2 in Figure 4(a). From the top-left to bottom right are a bicrystalline region, stacking faults and single crystalline region. The diffraction patterns corresponding to the three regions are shown in (b-d), representative of the bicrystal capping and nanowire (yellow box), stacking faults below the wire (red box) and single crystal GaAs nanomembrane (pink box).

S3 Details of modeling and genetic algorithm

The model used here is similar to that used to model dopant incorporation in vapor-liquid-solid (VLS) nanowires.[6,7] The dependence of the effective solubility of Si on Al concentration is represented in an effective partition coefficient k, defined as the ratio between the bulk and surface solubility ($k(\theta_{\mathrm{Al}}) \equiv c_{\mathrm{Si}}^{\mathrm{bulk}}/c_{\mathrm{Si}}^{\mathrm{surf}}$), which takes the form $k(\theta_{\mathrm{Al}}) = c_0 + c_1\theta_{\mathrm{Al}}$, where $c_0 = c_{\mathrm{Si}}^{\mathrm{GaAs}}/c_{\mathrm{Si}}^{\mathrm{surf}}$, and c_1 captures the change in the partition coefficient due to the presence of Al. We then solve for the partition coefficient for GaAs and the Al-induced relative enhancement k/c_0 that best describes the observed Si distribution (black line in Figure 3(b)).

S3.1 Description of empirical model

The dopant incorporation rate is given by[8]

$$\frac{d\theta_{\mathrm{surf}}}{dt} = J - k_{\mathrm{out}}\theta_{\mathrm{surf}} - I_{\mathrm{d}}, \tag{1}$$

where θ_{surf} is the Si concentration at the growth surface in cm^{-3}, J is the incident dopant flux, k_{out} is the desorption rate coefficient, and I_d is the net incorporation rate across the growth surface. In our simple model, I_d is a product of an Al-concentration dependent partition coefficient $g(\theta_{\mathrm{Al}})$ and the surface Si concentration $\theta_{\mathrm{surf}}(x)$:

$$I_{\mathrm{d}} = g(\theta_{\mathrm{Al}})\theta_{\mathrm{surf}}(x), \tag{2}$$

which is valid when the surface coverage of Si is much less than a monolayer. Further assuming that desorption can be neglected, the dopant incorporation rate simplifies to:

$$\frac{d\theta_{\mathrm{surf}}}{dt} = J - g(\theta_{\mathrm{Al}})\,\theta_{\mathrm{surf}}(x) \tag{3}$$

The time evolution of surface Si concentration will thus be reflected in the spatial distribution. By assuming that the nanowire growth rate v is constant, we can eliminate the time dependence of the differential equation and write

$$\frac{d\theta_{\mathrm{surf}}}{dx}(x) = J/v - k(\theta_{\mathrm{Al}}(x))\,\theta_{\mathrm{surf}}(x), \tag{4}$$

where $k(\theta_{\mathrm{Al}}) \equiv g(\theta_{\mathrm{Al}})/v$, and θ_{Al} is the bulk Al concentration measured by APT.

Given that changes in the relevant chemical potentials governing dopant incorporation can be linearized for sufficiently small perturbations in composition, our simple empirical model assumes the Si incorporation rate is proportional to the Si surface concentration (as noted above), and that the incorporation rate increases linearly with Al surface concentra-

tion. We represent these dependencies in the partition coefficient k, defined as the ratio between the bulk and surface solubility ($k = c_{\mathrm{Si}}^{\mathrm{bulk}}/c_{\mathrm{Si}}^{\mathrm{surf}}$), which takes the form

$$k(\theta_{\mathrm{Al}}) = c_0 + c_1\theta_{\mathrm{Al}} + \mathcal{O}(\theta_{\mathrm{Al}}^2), \tag{5}$$

where $c_0 = c_{\mathrm{Si}}^{\mathrm{GaAs}}/c_{\mathrm{Si}}^{\mathrm{surf}}$, and c_1 captures the change in the partition coefficient due to the presence of Al. We then solve for the partition coefficient of Si on pure GaAs (c_0), and the Al-induced relative enhancement k/c_0 that best describes the observed Si distribution, which can be defined as:

$$\theta_{\mathrm{bulk}}(x) = k(\theta_{\mathrm{Al}}(x))\,\theta_{\mathrm{surf}}(x) \tag{6}$$

The physical picture of the Al-enhancement in Si incorporation is presented in Figure 3(c) of the main text. In Figure 3(c)i), excess Si accumulates on the surface of the growing nanowire after the Si shutter is opened, leading to a gradual increase in Si incorporation (purple region in Figure 3(b)). This picture follows from from Eq. (4) - (6) in the absence of an Al flux, in which case k can be simplified to $k(\theta_{\mathrm{Al}}) = c_0$. Substituting k into Eq. (4), we find that $\frac{d\theta_{\mathrm{surf}}}{dx}(x) = \frac{J}{v} - c_0\theta_{\mathrm{surf}}(x) \approx \frac{J}{v}$, and substituting k into Eq. (6), we find that $\theta_{\mathrm{bulk}} = c_0\,\theta_{\mathrm{surf}}$. Combining these equations, $\theta_{\mathrm{bulk}} = (c_0\,\frac{J}{v})\,x \equiv l\,x$, where the slope l can be directly derived from the increase in Si concentration upon opening the shutter, again assuming that the coverage is much less than a monolayer. When the Al shutter is opened (blue region in Figure 3(b)), the Si begins to incorporate more rapidly, creating a spike in the Si concentration. As the excess surface Si becomes depleted, the incorporated Si concentration decreases rapidly, as observed in Figure 3(c)(ii) $\rightarrow$ (iii). Because the Si shutter closes when the Al shutter is opened, $\frac{J}{v} = 0$ in this region.

Therefore, change in surface Si concentration as a function of position can be written as:

$$\frac{d\theta_{\rm surf}}{dx}(x) = \begin{cases} l/c_0 - c_0\theta_{\rm surf}, & x <= x_{\rm Si}, \\ -(c_0 + c_1\theta_{\rm Al})\theta_{\rm surf}, & x > x_{\rm Si}. \end{cases} \tag{7}$$

where $x_{\rm Si}$ is the position that Si flux stops.

S3.2 Description of genetic algorithm

Since the master equation Eq. (7) is not explicitly differentiable by the fitting parameters, optimization methods based on gradient descent are not applied here. Instead, we used a genetic algorithm[9] to find the best fit of the measured profile of Si bulk concentration. Briefly, a genetic algorithm is an algorithm in which the solution is found by sequentially evolving a population of potential solutions to minimize the loss function. Here, we define the loss as a weighted $L1$ norm: $L(\theta, \hat{\theta}) = \sum_i |\hat{\theta}_{\rm i} - \theta_{\rm i}| \frac{\theta_i}{\sum_i \theta_i}$. In each iteration, the 20% of the population of parameters c_0, c_1, $x_{\rm Si}$ that gives the smallest loss are selected as "elite", while a "mutation" is applied to the remaining 80% of the population, by adding a vector from a Gaussian distribution to the "elites". The new population of parameters is filled back into the loop and evaluated with the same loss function. The iteration is terminated when the distribution of each parameter has converged and the loss does not decrease further.

The fit parameters in Figure 3(b) also describe well the Si profile on the right-hand facet as shown in Figure S7.

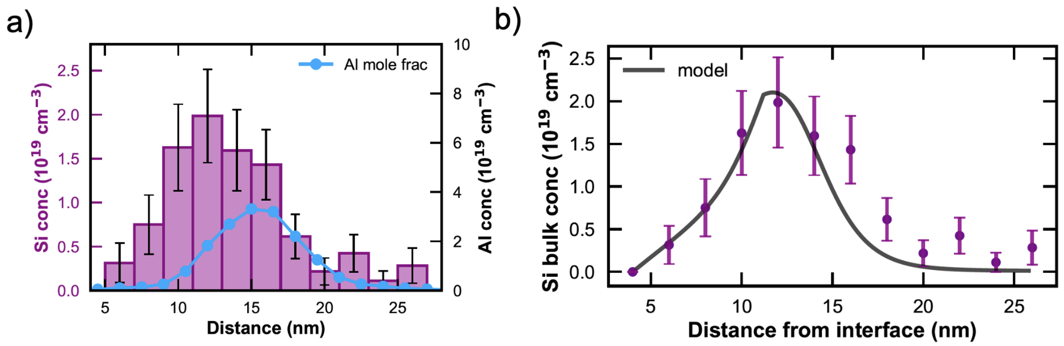

Fig. S7: (a) Si concentration profile together with a plot of Al mole fraction extracted from a sub-region across the right oblique facet of Sample-2. (b) Fitted Si profile applying the same c_0 and c_1 values used for fitting the profile of the left facet.

S4 Finite element modeling

S4.1 Details of finite element simulation

While the simulation is based on the composition and morphology extracted from APT and TEM analyses, it differs from the structure of Samples 1 and 2 in two ways. First, the nanomembranes analyzed in by APT analysis were ∼50 nm in width so that the entire upper faceting supporting the InGaAs nanowire could be captured within the APT field of view. The simulated nanowires are positioned on wider (∼80 nm) nanomembranes, which is necessary for this approach to achieve a sufficiently high In concentration and the desired electron localization.[10] The nanowire cross-section in Figure 4(d) is similar to that demonstrated in a previous study.[1] Second, the APT samples were grown with a high Si concentration to improve the signal-to-noise of the dopant mapping, whereas the simulations use a lower active Si concentration of 2×10^{18} cm^{-3}. Unnecessarily high Si doping can result in dopant compensation, strain, clustering, and additional carrier scattering,[11–13] as well as carrier accumulation near the nanowire-nanomembrane interface (Supporting Information Figure S8). The key experimental advance reported here is the localization of the Si dopants with an AlGaAs layer to create a dopant-free spacer layer, which remains feasible at lower doping

levels. Finally, the 2 nm AlGaAs layer and the thicker GaAs layer in the simulation are representative of the experimentally measured structure.

S4.2 Choice of the GaAs spacer thickness

A GaAs spacer layer was used to separate dopants from the nanowire channel. The thin AlGaAs trapping layer and the GaAs spacer layer diminish electron transfer to the InGaAs wire, so their combined thicknesses must be chosen to enable sufficient electron accumulation in the nanowire. The simulations in Figure S10 show that a 2 nm trapping layer and 3 nm spacer enable electron accumulation in the nanowire.

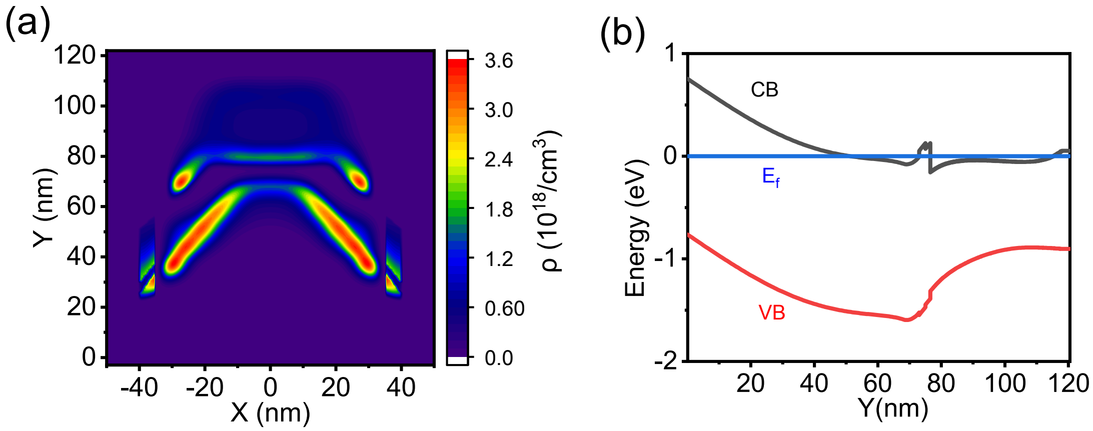

Fig. S8: (a) NextNano simulation of electron density map of a highly doped sample. Doping concentration is $8 \times 10^{18}/\mathrm{cm}^3$. (b) Band profiles taken along the y-axis at $x = 0$ in panel (a). Band bending in the membrane region increases more towards the doped region, resulting in the Fermi level staying above the conduction band edge.

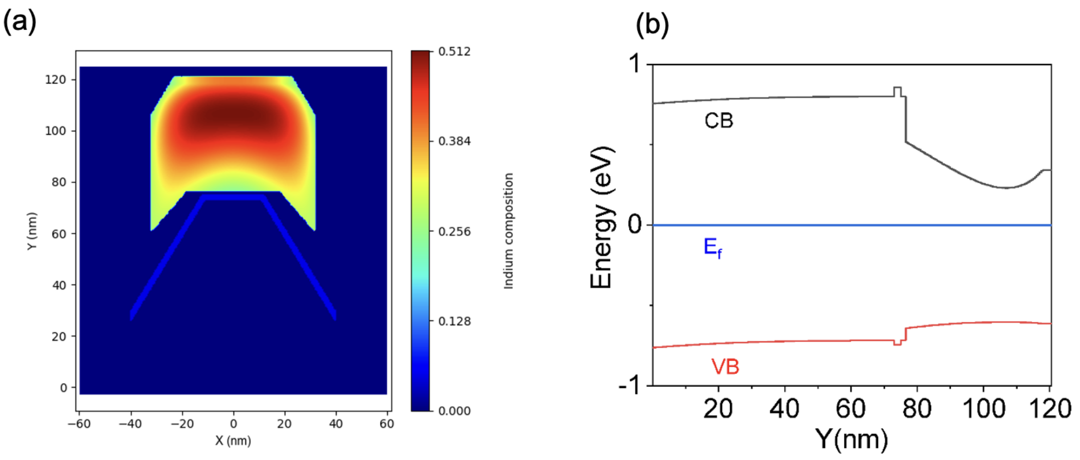

Fig. S9: (a) In concentration in the $In_xGa_{1-x}As$ nanowire used in the simulation with an undoped support. The In concentration was chosen based on our previous study.[1] (b) Band diagram taken along the y-axis at $x = 0$ in panel (a). The Fermi level stays below the conduction band edge.

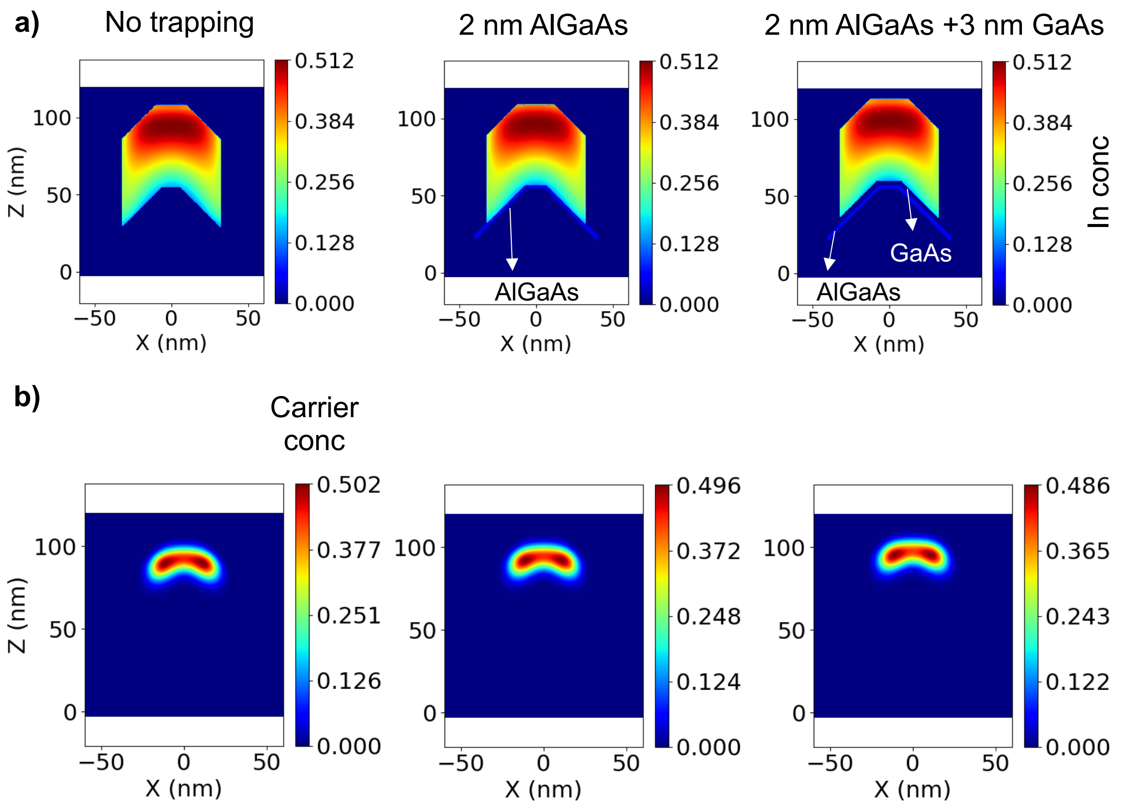

Fig. S10: (a) Geometry of the nanowire, AlGaAs trapping layer and GaAs spacer, with increasing thickness of spacer from left to right. (b) Corresponding NextNano simulation of the electron density in the nanowire.

References

(1) Friedl, M.; Cerveny, K.; Huang, C.; Dede, D.; Samani, M.; Hill, M. O.; Morgan, N.; Kim, W.; Güniat, L.; Segura-Ruiz, J.; Lauhon, L. J.; Zumbühl, D. M.; Fontcuberta i Morral, A. Remote Doping of Scalable Nanowire Branches. *Nano Letters* **2020**, *20*, 3577–3584.

(2) Miller, M. K.; Russell, K. F.; Thompson, K.; Alvis, R.; Larson, D. J. Review of atom probe FIB-based specimen preparation methods. *Microscopy and microanalysis* **2007**, *13*, 428–436.

(3) Thompson, K.; Lawrence, D.; Larson, D.; Olson, J.; Kelly, T.; Gorman, B. In situ site-specific specimen preparation for atom probe tomography. *Ultramicroscopy* **2007**, *107*, 131–139.

(4) Langford, R.; Rogers, M. In situ lift-out: steps to improve yield and a comparison with other FIB TEM sample preparation techniques. *Micron* **2008**, *39*, 1325–1330.

(5) Jublot, M.; Texier, M. Sample preparation by focused ion beam micromachining for transmission electron microscopy imaging in front-view. *Micron* **2014**, *56*, 63–67.

(6) Dufouleur, J.; Colombo, C.; Garma, T.; Ketterer, B.; Uccelli, E.; Nicotra, M.; Fontcuberta i Morral, A. P-doping mechanisms in catalyst-free gallium arsenide nanowires. *Nano Letters* **2010**, *10*, 1734–1740.

(7) Perea, D. E.; Hemesath, E. R.; Schwalbach, E. J.; Lensch-Falk, J. L.; Voorhees, P. W.; Lauhon, L. J. Direct measurement of dopant distribution in an individual vapour–liquid–solid nanowire. *Nature Nanotechnology* **2009**, *4*, 315–319.

(8) Greene, J.; Barnett, S.; Rockett, A.; Bajor, G. Modeling of dopant incorporation, segregation, and ion/surface interaction effects during semiconductor film growth by

molecular beam epitaxy and plasma-based techniques. *Applications of Surface Science* **1985**, *22-23*, 520–544.

(9) Huntington, M. D.; Lauhon, L. J.; Odom, T. W. Subwavelength lattice optics by evolutionary design. *Nano Letters* **2014**, *14*, 7195–7200.

(10) Friedl, M. et al. Template-Assisted Scalable Nanowire Networks. *Nano Letters* **2018**, *18*, 2666–2671.

(11) Ketterer, B.; Mikheev, E.; Uccelli, E.; Fontcuberta i Morral, A. Compensation mechanism in silicon-doped gallium arsenide nanowires. *Applied Physics Letters* **2010**, *97*, 223103.

(12) Domke, C.; Ebert, P.; Heinrich, M.; Urban, K. Microscopic identification of the compensation mechanisms in Si-doped GaAs. *Phys. Rev. B* **1996**, *54*, 10288–10291.

(13) Venkatasubramanian, R.; Patel, K.; Ghandhi, S. Compensation mechanisms in n^+-GaAs doped with silicon. *Journal of Crystal Growth* **1989**, *94*, 34–40.
